# Complete Genome Sequence of *Staphylococcus aureus* MCRF184, a Necrotizing Fasciitis-Causing Methicillin-Sensitive Sequence Type 45 *Staphylococcus* Strain

**DOI:** 10.1128/genomeA.00374-16

**Published:** 2016-05-12

**Authors:** Vijay Aswani, Bob Mau, Sanjay K. Shukla

**Affiliations:** a Department of Internal Medicine and Pediatrics, Marshfield Clinic, Marshfield, Wisconsin, USA; b Genome Center of Wisconsin, University of Wisconsin, Madison, Wisconsin, USA; c Center for Human Genetics, Marshfield Clinic Research Foundation, Marshfield, Wisconsin, USA

## Abstract

We report here the complete genome sequence of a highly virulent methicillin-sensitive *Staphylococcus aureus* strain*,* MCRF184, belonging to sequence type 45. This staphylococcal strain was isolated from a surgical biopsy specimen from a patient with necrotizing fasciitis.

## GENOME ANNOUNCEMENT

*Staphylococcus aureus* is a remarkable pathogen with emerging virulence. It is an uncommon cause of necrotizing fasciitis (1). However, the virulence associated with certain *S. aureus* strains and their increased ability to cause necrotizing fasciitis compared to others is not understood. To gain a better understanding of the overall pathogenesis and identify novel targets for new chemotherapeutic agents, researchers have been sequencing particularly virulent strains of *S. aureus* (2). In this paper, we report the complete annotated genome sequence obtained for *S. aureus* MCRF184, a strain isolated from the wounds of a 72-year-old diabetic male who suffered an above-the-knee amputation on his left leg due to necrotizing fasciitis (3).

The MCRF184 genome was sequenced by both a shotgun (single-end) and paired-end library approach. The shotgun and paired-end libraries were prepared and sequenced by using the GS TLX Titanium and GS FLX standard kits and protocols (Roche), respectively. The average read lengths for the shotgun and paired-end libraries were 171 bp and 300 bp, respectively. The 454 data were assembled using the gsAssembler program (version 2.0; Roche), which produced an overall depth of coverage between 25 and 30× for a 2.66-Mbp genome (70 to 84 Mbp of input sequence).

Overall, the assembly generated 233 large (≥500 bp) contigs spanning a total length of 2.66 Mbp. The paired-end information was then used to arrange 49 contigs into 19 scaffolds (total length of scaffolds, 2.66 Mbp). The two largest scaffolds, at 1.32 Mbp and 1.03 Mbp, account for 88% of this length. Further, they were assembled into 15 single contig scaffolds and 4 multicontig scaffolds (incorporating 33 contigs) representing the entire genome. The 33 contig sequences from MCRF184 were reordered in progressiveMauve (4) using reference strain *S. aureus* MRSA252 (accession no. BX571856). Transfer RNAs and ribosomal RNAs were predicted by using Rfam (5). Putative open reading frames (ORFs) of the MCRF184 genome were predicted by Glimmer (6) and from a progressiveMauve (4) alignment using reference strain *S. aureus* MRSA252 (accession no. BX571856). The final assembly and annotation were done in Geneious version 9.1.2 (7).

The MCRF184 genome contains 2,664,662 bp, with a G+C content of 32.9%. The genome contains 2,569 open reading frames, including 59 tRNA genes, 1 transfer-messenger RNA (tmRNA), and 16 rRNAs.

The initial characterization of the genome using multilocus sequence typing (MLST) analysis (8) revealed *S. aureus* MCRF184 to be a member of sequence type 45 (ST45), a USA600 strain, and it lacked the staphylococcal chromosomal cassette, confirming its methicillin-susceptible phenotype.

### Nucleotide sequence accession numbers.

The complete genome sequence of *S. aureus* MCRF184 has been deposited at DDBJ/EMBL/GenBank under the accession no. CP014791. The version described in this paper is version CP014791.1. *S. aureus* MRCF184 belongs to BioProject no. PRJNA39571 and BioSample no. SAMN02953006.

## References

[1] Miller LG, Perdreau-Remington F, Rieg G, Mehdi S, Perlroth J, Bayer AS, Tang AW, Phung TO, Spellberg B 2005 Necrotizing fasciitis caused by community-associated methicillin-resistant *Staphylococcus aureus* in Los Angeles. N Engl J Med 352:1445–1453. doi:10.1056/NEJMoa042683. 15814880

[2] Takeuchi F, Watanabe S, Baba T, Yuzawa H, Ito T, Morimoto Y, Kuroda M, Cui L, Takahashi M, Ankai A, Baba S, Fukui S, Lee JC, Hiramatsu K 2005 Whole-genome sequencing of *Staphylococcus haemolyticus* uncovers the extreme plasticity of its genome and the evolution of human-colonizing staphylococcal species. J Bacteriol 187:7292–7308. doi:10.1128/JB.187.21.7292-7308.2005. 16237012 PMC1272970

[3] Morgan WR, Caldwell MD, Brady JM, Stemper ME, Reed KD, Shukla SK 2007 Necrotizing fasciitis due to a methicillin-sensitive *Staphylococcus aureus* harboring an enterotoxin gene cluster. J Clin Microbiol 45:668–671. doi:10.1128/JCM.01657-06. 17166962 PMC1829043

[4] Darling AE, Mau B, Perna NT 2010 progressiveMauve: multiple genome alignment with gene gain, loss and rearrangement. PLoS One 5:e11147. doi:10.1371/journal.pone.0011147. 20593022 PMC2892488

[5] Nawrocki EP, Burge SW, Bateman A, Daub J, Eberhardt RY, Eddy SR, Floden EW, Gardner PP, Jones TA, Tate J, Finn RD 2015 Rfam 12.0: updates to the RNA families database. Nucleic Acids Res 43:D130–D137 doi:10.1093/nar/gku1063. 25392425 PMC4383904

[6] Delcher AL, Harmon D, Kasif S, White O, Salzberg SL 1999 Improved microbial gene identification with Glimmer. Nucleic Acids Res 27:4636–4641. doi:10.1093/nar/27.23.4636. 10556321 PMC148753

[7] Kearse M, Moir R, Wilson A, Stones-Havas S, Cheung M, Sturrock S, Buxton S, Cooper A, Markowitz S, Duran C, Thierer T, Ashton B, Meintjes P, Drummond A 2012 Geneious basic: an integrated and extendable desktop software platform for the organization and analysis of sequence data. Bioinformatics 28:1647–1649. doi:10.1093/bioinformatics/bts199. 22543367 PMC3371832

[8] Enright MC, Day NP, Davies CE, Peacock SJ, Spratt BG 2000 Multilocus sequence typing for characterization of methicillin-resistant and methicillin-susceptible clones of *Staphylococcus aureus*. J Clin Microbiol 38:1008–1015. 10698988 10.1128/jcm.38.3.1008-1015.2000 PMC86325

